# Advanced Material-Ordered Nanotubular Ceramic Membranes Covalently Capped with Single-Wall Carbon Nanotubes

**DOI:** 10.3390/ma11050739

**Published:** 2018-05-07

**Authors:** Samer Al-Gharabli, Eyad Hamad, Munib Saket, Ziad Abu El-Rub, Hassan Arafat, Wojciech Kujawski, Joanna Kujawa

**Affiliations:** 1 Pharmaceutical and Chemical Engineering Department, German Jordanian University, Amman 11180, Jordan; munib.saket@gju.edu.jo (M.S.); ziad.abuelrub@gju.edu.jo (Z.A.-E.R.); 2 Biomedical Engineering Department, German Jordanian University, Amman 11180, Jordan; eyad.hamad@gju.edu.jo; 3 Center for Membrane and Advanced Water Technology, Department of Chemical Engineering, Khalifa University of Science and Technology, P.O. Box 54224, Abu Dhabi, UAE; hassan.arafat@ku.ac.ae; 4 Faculty of Chemistry, Nicolaus Copernicus University in Toruń, 7 Gagarina Street, 87-100 Toruń, Poland; wojciech.kujawski@umk.pl

**Keywords:** ordered ceramic membrane, single-wall carbon nanotubes, functionalization, tribology

## Abstract

Advanced ceramic materials with a well-defined nano-architecture of their surfaces were formed by applying a two-step procedure. Firstly, a primary amine was docked on the ordered nanotubular ceramic surface via a silanization process. Subsequently, single-wall carbon nanotubes (SWCNTs) were covalently grafted onto the surface via an amide building block. Physicochemical (e.g., hydrophobicity, and surface free energy (SFE)), mechanical, and tribological properties of the developed membranes were improved significantly. The design, preparation, and extended characterization of the developed membranes are presented. Tools such as high-resolution transmission electron microscopy (HR-TEM), single-area electron diffraction (SAED) analysis, microscopy, tribology, nano-indentation, and Raman spectroscopy, among other techniques, were utilized in the characterization of the developed membranes. As an effect of hydrophobization, the contact angles (CAs) changed from 38° to 110° and from 51° to 95° for the silanization of ceramic membranes 20 (CM20) and CM100, respectively. SWCNT functionalization reduced the CAs to 72° and 66° for ceramic membranes carbon nanotubes 20 (CM-CNT-20) and CM-CNT-100, respectively. The mechanical properties of the developed membranes improved significantly. From the nanotribological study, Young’s modulus increased from 3 to 39 GPa for CM-CNT-20 and from 43 to 48 GPa for pristine CM-CNT-100. Furthermore, the nanohardness increased by about 80% after the attachment of CNTs for both types of ceramics. The proposed protocol within this work for the development of functionalized ceramic membranes is both simple and efficient.

## 1. Introduction

Porous materials, including nanoporous membranes, have been known to scientists and engineers for several decades. Porous membranes are used in various novel applications such as adsorption, catalysis, molecular separation, biosensing, and energy storage, as well as in drug delivery and template synthesis [1,2,3,4,5]. Two crucial characteristics of porous membrane performance are the membrane’s flux and selectivity. Various nanomaterials, such as inorganic oxides, polymers, carbon, metals, zeolites, and metallo-organic composites [3,6,7,8,9], have been investigated for the fabrication of porous membranes. Methods such as ion track-etching or lithography are known to produce membranes with well-defined cylindrical pores [3,4,10,11]. Nanomaterials that have well-defined pore structures find efficient applications in gas and water purification and separation technologies (e.g., carbon dioxide removal from natural gas, distillation, medical applications, etc.).

In addition to the physical features of the membrane, the membrane’s susceptibility to chemical modification also plays an important role in defining its selectivity and biofouling resistance potential. In this context, carbon nanotubes (CNTs) are strong candidates for membrane materials because of their superior properties and chemical affinity for anchoring additional moieties [12,13,14,15]. In addition, CNTs possess unique electrical and thermal properties and extraordinary mechanical strength [2,3,7,15]. Therefore, CNTs are used in a broad spectrum of applications, such as nanofiltration [16], Li-ion secondary batteries [17], supercapacitors [18,19], hydrogen storage in fuel cells, and sensors [2,20,21,22,23]. CNTs are credited with exceptional mass transport properties because of the atomic smoothness of their surfaces, extreme frictionless graphitic interfaces, and molecular ordering phenomena [24]. Consequently, CNT-based membranes (microfabricated membranes with aligned CNTs of diameters less than 2 nm) used for separation processes are reported to produce higher flux by almost 4–5 orders of magnitude in comparison with other membrane materials of comparable pore sizes. This phenomenon was attributed to the extreme frictionless graphitic interfaces as well as defect-free, smooth walls of CNTs [1]. Moreover, CNT membranes are analogous to biological membrane channels, for example, aquaporin-1 [2]. It has been recently reported that ceramic composite membranes grafted with CNTs via chemical vapor deposition (CVD) not only provided higher water flux, but also exhibited high oil rejection and selectivity for copper ion removal [25]. Biomimetic membranes have also been designed by employing CNTs in the regular pores [2,26,27,28]. However, the physical deposition of CNTs on membrane surfaces could also result in undesirable and uncontrolled leaching of CNTs from the surfaces.

In this work, single-wall carbon nanotubes (SWCNTs) were covalently attached to the surface of ceramic alumina membranes with well-defined parallel cylindrical pores prepared via an electron-beam lithography screening method. The specific method of covalent linkage of the CNTs mitigates the issues with CNT leaching encountered in other deposition techniques. This functionalization led to the modification of the membranes’ physicochemical properties, which were thoroughly characterized in this work.

## 2. Materials and Methods 

### 2.1. Materials

Ceramic alumina membranes (pore sizes of 20 and 100 nm) were purchased from i3 Membrane GmbH (Dresden, Germany). (3-Aminopropyl)triethoxysilane (T-NH_2_), 1-methyl-2-pirolodine, *N*,*N*-dimethylformamide, *O*-(benzotriazol-1-yl)-*N*,*N*,*N*′,*N*′-tetramethyluronium tetrafluoroborate (TBTU), *N*,*N*-diisopropylethylamineamine, dichloromethane (DCM), xylene, toluene, tetrahydrofuran, *n*-dodecane, cyclohexane, hexane, methanol (MeOH), and glycerol were purchased from Sigma-Aldrich (St. Louis, MO, USA). SWCNTs functionalized with 2.75 wt % carboxylic groups (SWCNT–COOH) with an outside diameter of 1–2 nm and a length of 5–30 µm were purchased from MKnano (Mississauga, ON, Canada).

### 2.2. Membrane Modification

The modification process of the ceramic membranes employed in this study generally consisted of two steps: a reaction with T-NH_2_ and subsequent functionalization by SWCNT–COOH. The aim of the first stage was to covalently anchor the silane coupling agent to the pristine ceramic surface naturally rich in hydroxyl groups.

Prior to modification, samples of alumina ceramic membranes with pore sizes of 20 nm (labeled as CM-20) and 100 nm (labeled as CM-100) were washed with methanol, acetone, and water for 10 min in each solvent and subsequently dried in an oven at 70 °C for 12 h. Samples were then placed in 50 mL of 0.1 M T-NH_2_ in toluene at room temperature under an inert atmosphere. The membrane samples were shaken in a grafting solution for 3 h, removed, and washed with toluene, methanol, and DCM. The washing process was repeated five times. Modified membranes (labeled as CM-NH_2_-20 and CM-NH_2_-100) were subsequently dried in an oven at 70 °C for 12 h. Membranes were further functionalized with CNTs, whereby 10 mg of SWCNT–COOH was suspended in 50 mL of dry DCM by ultra-sonication at room temperature for 10 min. To generate the corresponding active ester, 2 mg of TBTU was then added to the sonicated mixture, followed by the addition of 1.0 µL of *N*,*N*-diisopropylethylamine (DIPEA). Subsequently, CM-NH_2_-20 or CM-NH_2_-100 was added to the reaction mixture and kept under sonication for another 3 h. Bath sonication was used. The resultant membranes were washed under sonication five times each with DCM, MeOH, and H_2_O, and then again with MeOH in sequence. The membranes were then dried in an oven at 70 °C for 3 h to obtain CNT-functionalized membranes, labeled as CM-CNT-20 and CM-CNT-100, corresponding to 20 and 100 nm pore sizes, respectively.

### 2.3. Membrane Characterization

Pristine and modified membranes were characterized by static and dynamic goniometric methods (Krüss Easy Drop Analyzer, Hamburg, Germany) using a constant volume (3 µL) of the testing liquid and equilibration time (5 s) at room temperature to determine the values of the apparent contact angle (CA) with ±0.5° accuracy. The following solvents were utilized as testing liquids: water (γ = 72.7 mN·m^−1^), glycerol (γ = 63.4 mN·m^−1^), 1-methyl-2-pirolodine (γ = 40.8 mN·m^−1^), *N*,*N*-dimethylformamide (γ = 37.1 mN·m^−1^), xylene (γ = 30.1 mN·m^−1^), toluene (γ = 28.5 mN·m^−1^), tetrahydrofuran (γ = 27.4 mN·m^−1^), n-dodecane (γ = 25.4 mN·m^−1^), cyclohexane (γ = 25.2 mN·m^−1^), and hexane (γ = 18.5 mN·m^−1^). 

Microscopic imaging was done using a Field Emission Scanning Electron Microscopy (FE-SEM) device (FEI Nova NanoSEM 650, Hillsboro, OR, USA). FE-SEM samples were sputtered with a 100 Å layer of gold to improve the conductivity and quality of the collected data.

The surface morphology was evaluated by Atomic Force Microscopy (AFM) imaging using the NanoScope MultiMode SPM System, NanoScope IIIa, and Quadrex controller (Veeco, St Ives, UK). The sample roughness was measured using the tip-scanning mode and was reported as a root-mean-squared (RMS) value. Using the same mode, the adhesion force (F_adh_) was evaluated as an average of 40 measurements. The scan size of the sample was 5 µm × 5 µm. In addition, the tribological measurements and nanohardness, as well as Young’s modulus, were also determined. A three-sided pyramid diamond cantilever (cantilever spring constant: 859 N/m) with a 60° apex angle was utilized. All samples were analyzed five times, and an average value was calculated (accuracy of ±4%). Ambient temperature was kept constant during all the experiments.

High-resolution transmission electron microscopy (HR-TEM) was performed using a Tecnai G2 F20 X-Twin (FEI Europe, Frankfurt/Main, Germany), by applying an accelerating voltage of 200 kV. Raman spectra were collected using a Witec Alpha 300 RAS (Ulm, Germany) with a 633 nm LASER light. The integration and accumulation times were 2–30 and 250 s, respectively.

## 3. Results and Discussion

### 3.1. Properties of the Pristine Membranes

**Figure 1:**
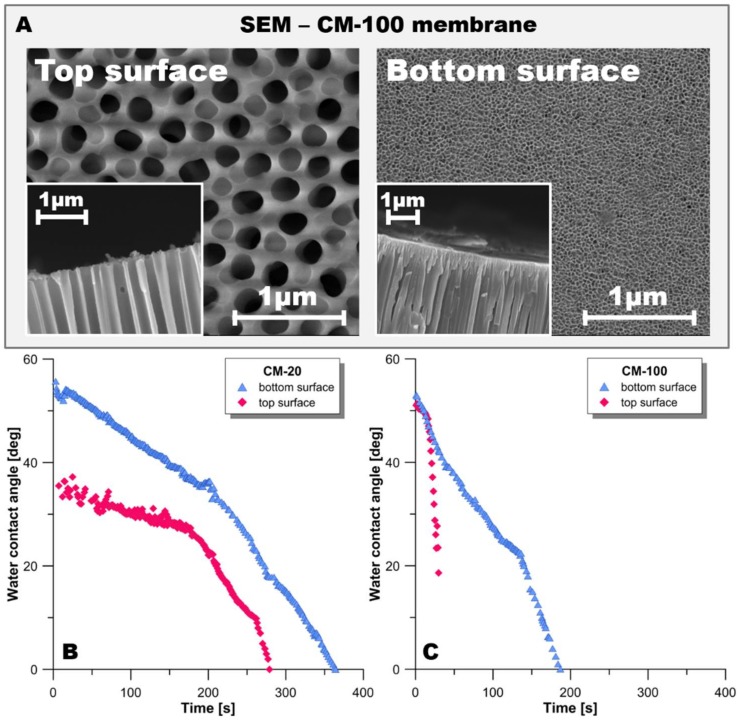
(**A**) SEM images of top and bottom sides of the pristine 100nm CM-100 membrane. Dynamic contact angle (CA) measurements on CM-20 (**B**) and CM-100 (**C**) membranes.

Pristine membranes were characterized using Scanning Electron Microscopy (SEM) and dynamic and static goniometric measurements. Tests were done on both sides of the membranes because of differences in morphology. From Figure 1A, it is evident that the top surface of the CM-100 membrane possessed large pore diameters when compared to the bottom surface. The top surface of CM-100 had a CA of 50 ± 5°, while the bottom surface had a CA of 55 ± 5°. In comparison, the CA of the top surface of CM-20 was smaller (35 ± 5°). The bottom surface of CM-20 had a CA (53 ± 5°) similar to the bottom surface of CM-100. Figure 1B,C depicts the dynamic CA measurements of the top and bottom surfaces of CM-20 and CM-100, respectively. For a given period of time, the sample with the greater reduction in the CA depicted a faster wetting rate. From Figure 1B,C, it is seen that both the top and bottom surfaces of CM-100 had a relatively faster rate of wetting in comparison with CM-20 surfaces. The top surface of CM-100 exhibited a 9-fold faster wetting rate than the top surface of CM-20, while the bottom surface of CM-100 had a wetting rate twice that of the bottom surface of CM-20. These results indicate that, although both membranes are hydrophilic, an initial difference in water soaking exists between them.

**Figure 2:**
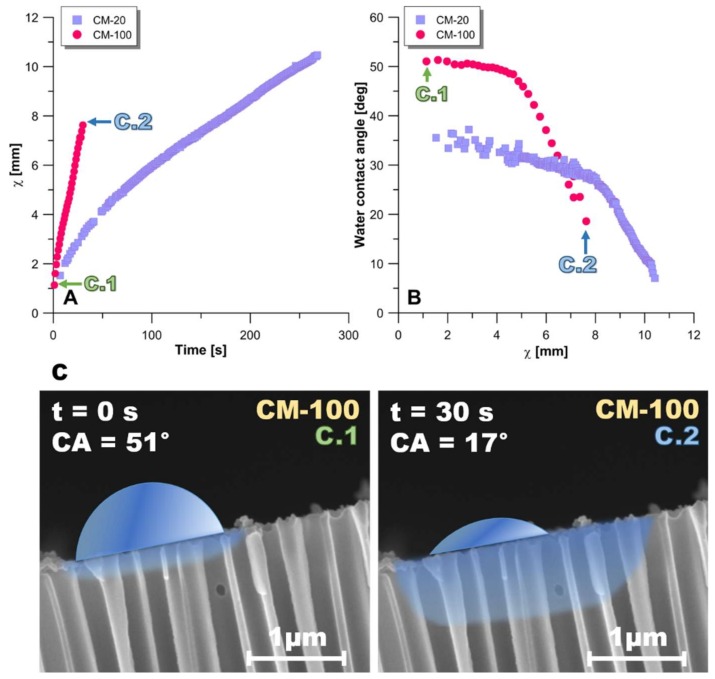
Behavior of water drop deposited on pristine membranes CM-20 and CM-100: (**A**) evolution of χ parameter as a function of time; (**B**) the correlation between χ and the apparent water contact angle (CA); (**C**) visualization of water drop behavior directly after deposition (**C.1**) and after 30 s (**C.2**).

The water wettability of the pristine membranes was evaluated by examining the profile of liquid penetration into the ceramic structure. Equation (1) was used for the calculation of the liquid penetration depth (X) (m) by employing the following parameters: pore size (d_p_) (m), liquid–vapor surface tension (Y_LV_) (mN·m^−1^), CA (deg), time (t) (s), and wetting liquid viscosity (η) (Pa·s). Using Equation (1) and the dynamic CA values from Figure 1, the depth of the liquid penetration (X) was calculated as illustrated in Figure 2.

(1) $$X=\sqrt{\frac{d_{p}\gamma_{\mathrm{LV}}t\cos(\mathrm{CA})}{4\eta }}$$

According to Lisovsky [26] and Mu et al. [29], a wetting liquid can spread on the particles and penetrate into the 3D mesh structure of ceramics in a few seconds, even under static conditions. However, in ceramic membranes, the spreading phenomenon is affected also by the membrane pore size (Figure 2). For the membrane with larger pores (CM-100), the water droplet penetrated into the membrane structure within 30 s of the first contact. The liquid penetration depth was around 8 mm (Figure 2A), while the apparent CA reduced from 51° to 17° (Figure 2B). A visualization of the water drop behavior on the surface of CM-100 is depicted in Figure 2C. In the case of the membrane with smaller pores (CM-20), after a contact time of 30 s, the liquid penetration depth was relatively shallower (3 mm), while the apparent CA decreased from 36° to 7° during water dispersion (Figure 2B). It is understood that, although CM-100 has a greater apparent CA, its wetting rate and liquid penetration depth are greater in comparison with the smaller-pore membrane CM-20.

### 3.2. Confirmation of Membrane Functionalization

**Figure 3:**
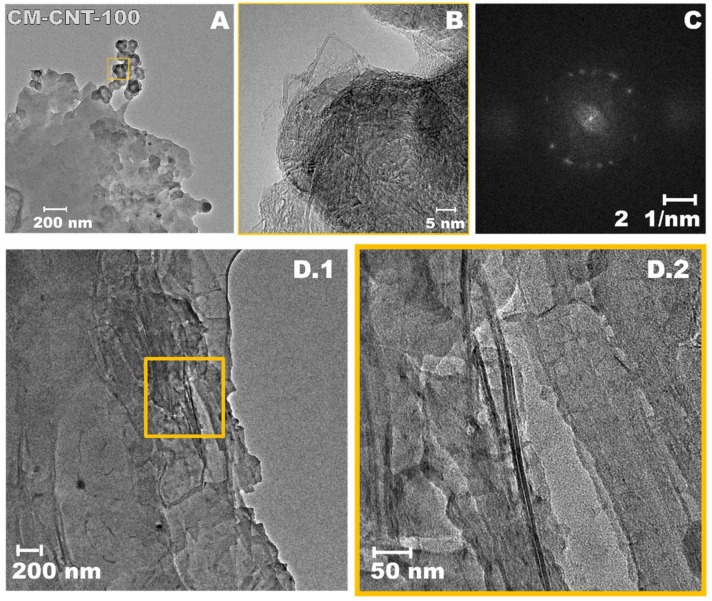
TEM images of modified CM-CNT-100 sample (**A**), with the yellow box in (**A**) magnified in (**B**), and SAED pattern (**C**). CNT located in pores of ceramic membrane. (**D.1**,**D.2**) Magnification.

**Figure 4:**
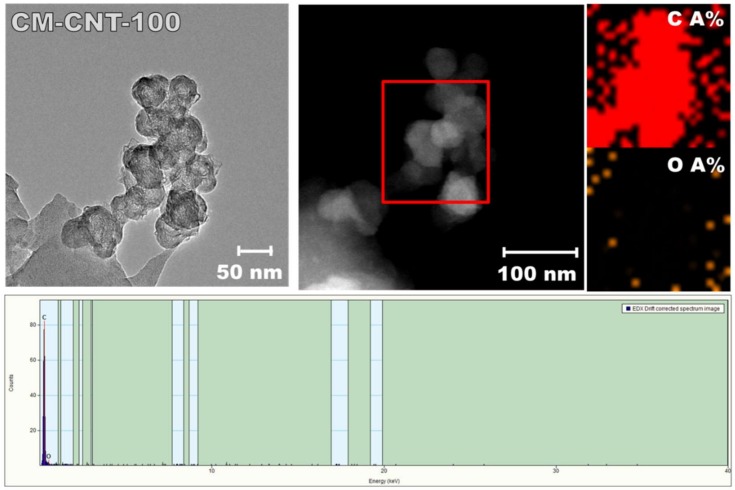
Energy-dispersive X-ray spectroscopy (EDX) analysis of TEM-modified membrane (CM-CNT-100): presence of carbon and oxygen.

Ceramic membrane modification was assessed by HR-TEM analysis (Figure 3). On the basis of Transmission Electron Microscopy (TEM) micrographs, the diameter of the SWCNTs was found to be in the range of 1–2 nm (Figure 3B). Figure 3 and Figure 4 show the attachment of SWCNTs to the membrane surface and even within the pores (deeper in the membrane structure). Moreover, in Figure 4C, the presence of dots on the single-area electron diffraction (SAED) pattern confirmed an increase in the crystal orientation degree of the new structure. This could have been caused by the partial alignment of SWCNTs on the ceramic surface (Figure 3D). The majority of the SWCNTs present inside the membrane were in a bundle form (Figure 4). Thus, different orientations were found (Figure 4C). However, the distance between parallel fringes was in the range from 0.25 to 0.30 nm. Figure 3B clearly demonstrates isolated SWCNTs also attached to the surface of the membrane.

**Figure 5:**
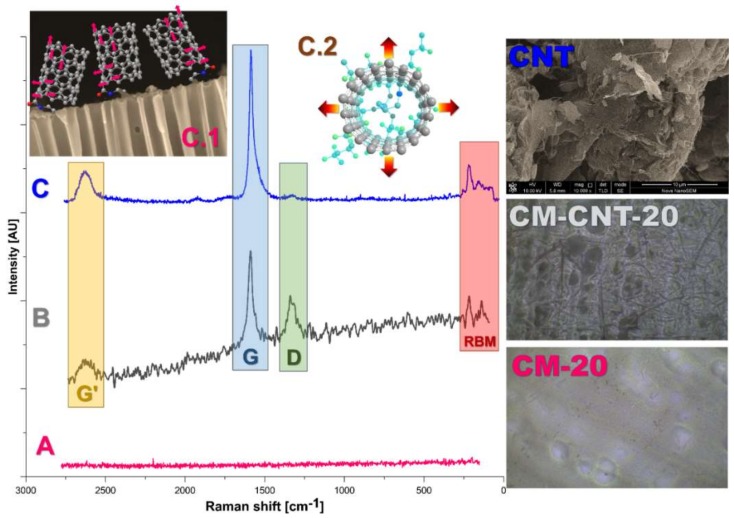
Raman spectra of pristine (CM-20) (**A**) and modified (CM-CNT-20) (**B**) membranes and pure single-wall carbon nanotubes (SWCNTs) (**C**): (**C.1**) G-band explanation consists of G^+^ (vibrations along the nanotube axis—longitudinal optical phonon mode) and G^−^ (vibrations along the circumferential direction of the nanotube—transverse optical phonon mode); (**C.2**) radial breathing mode (RBM) band.

Raman spectroscopy is a powerful tool in identifying several properties of CNT samples such as chirality, size distribution, and architecture, as well as purity [30,31,32]. Figure 5 depicts the Raman spectra of pristine and modified ceramic membranes. In the case of pristine membrane, no band was observed because Al_2_O_3_ in gamma form is not active in Raman spectroscopy [33]. However, the presence of various bands was noticed in the modified membranes after the chemical attachment of CNTs (Figure 5). The most intensive peak found at 1591 cm^−1^ was associated with the C–C stretching bonds between the two dissimilar carbon atoms in the graphite plane (Figure 5C1). The CNTs exhibited two main characteristics in the first-order Raman spectra: (a) a G-band referring to the tangential mode vibrations, and (b) the radial breathing mode (RBM). The Raman spectra of the modified membranes, in Figure 5, have the G-band [34] with its two corresponding components, G^+^ and G^−^, which are related to the vibrations along the length of the nanotube axis (longitudinal optical phonon mode) and vibrations along the circumferential direction of the nanotube (transverse optical phonon mode), respectively [15]. The RBM peaks (Figure 5C2) were recorded at 219 and 152 cm^−1^ of Raman shift, which are significant to SWCNT and cannot be noticed in the graphite plate (Figure 5B). It has been reported that the RBMs are associated with coherent movement of carbon atoms in the radial direction [35]. By measuring the RBM frequency, the diameter distribution of the CNTs could be determined. The RBM possessed two components: one appearing as a shoulder shifted in the direction of higher frequencies corresponding to nanotubes in a bundle environment, and another, generally more intense, associated with isolated nanotubes. In the case of SWCNTs, the wavelength of the Raman shift of the RBM was inversely proportional to the SWCNT dimension. Furthermore, on the basis of the RBM Raman shift, the dimension of isolated (Equation (2)) and bundled (Equation (3)) SWCNTs could be determined, according to the following formulas [35]. In the present work, the calculated diameters of isolated and bundled SWCNTs were 1.75 and 1.13 nm, respectively.

(2) ω=248/d

(3) ω=10+(234/d)

For a qualitative analysis of the defects in the sample, the intensities of the D- and G-bands were compared. Generally, for high-quality SWCNTs, which are defect- and amorphous-carbon-free, the ratio of the D- and G-band intensities should be less than 2% [35,36,37]. In the present work, the D/G ratio of the pure SWCNTs was 2.7%, indicating the almost-defect-free SWCNTs. Upon covalently grafting SWCNTs on the ceramic membrane, the D/G ratio increased by 40% for the modified membrane. In addition, intensive G’ peaks located in the higher-frequency region were observed at 2626 cm^−1^ for CM-CNT-20 and at 2631 cm^−1^ for pure SWCNTs (Figure 5C,B). The G’ peak is an intrinsic feature of graphite and CNTs that can be observed in totally defect free SWCNT samples for which the D band will be absent [15,38]. The formation of a chemically stable covalent bond between the ceramic membrane and SWCNTs can be assessed by the increased shift and intensity of the D-band. The increased Raman shift for the modified membrane CM-CNT-20 (1343 cm^−1^) in comparison with the pure SWCNTs (Raman shift = 1324 cm^−1^) might have been related to the partial coupling of the active sites, indicating a formation of stable covalent bonds [38,39] between the ceramic support and SWCNTs (Figure 5C1).

### 3.3. Properties of the Modified Membranes

#### 3.3.1. Surface Properties

**Figure 6:**
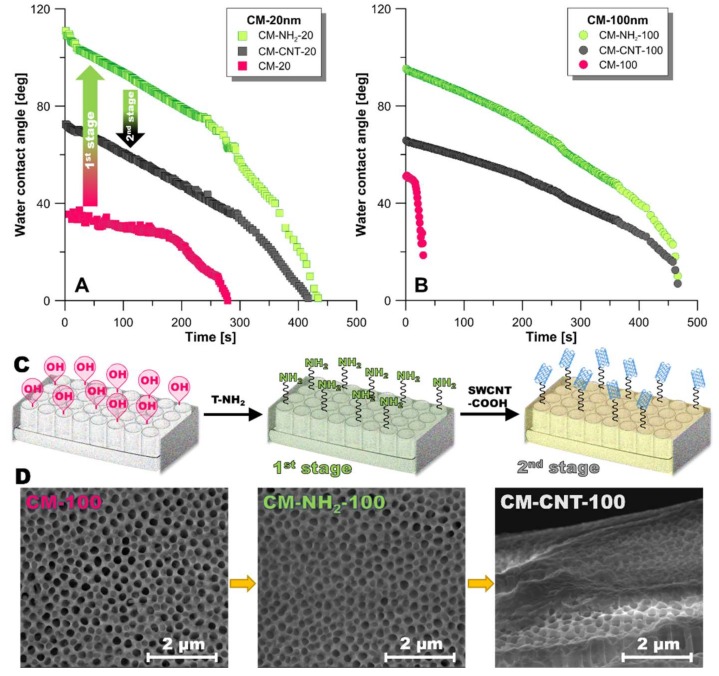
Dynamic contact angle (CA) measurements for modified samples of CM-20 (**A**) and CM-100 (**B**); (**C**) SEM images of the membrane after first (with 3-aminopropyl)triethoxysilane—T-NH_2_) and second step (with carbon nanotubes—CNTs) of modification.

Ceramic membranes are naturally hydrophilic because of the presence of hydroxyl groups (–OH) on the surface including the membrane pores [40,41]. SEM images illustrate the presence of a semi-transparent layer resulting from the first stage of the modification process (Figure 6C). The effectiveness of the grafting with T-NH_2_ was confirmed by the positive result of the Kaiser test, a selective test towards primary amines. Upon modification, the surfaces of both pristine membranes switched from being hydrophilic to being hydrophobic (Figure 6A,B), and the wetting resistance was also altered. It was noticed that, in the case of the CM-20 membrane, the CA increased from 35° to 110° for CM-NH_2_-20. For CM-100, the CA increased from 50° to 95° for CM-NH_2_-100. These differences in CA were associated only with the evaporation of the test liquid, as confirmed by a constant drop base diameter and gradual changes in volume. The water repellency in both modified membranes was significantly increased. For both amine-functionalized or silanized membranes, no water penetration into the membranes was observed (Figure 6), unlike that observed with the pristine membranes (Figure 1C).

The modified samples were then further functionalized by chemically anchoring SWCNTs. Upon functionalization with CNTs, the apparent CAs for CM-CNT-20 and CM-CNT-100 dropped to 72° and 66°, respectively. The reduction in the CAs can be correlated to the presence of hydrophilic SWCNTs. Thus, the final modified membrane was a multilayer hybrid hydrophilic/hydrophobic composite. This is advantageous because the particles that cause fouling in water treatment are mostly hydrophobic. Thus, one approach to reducing fouling is to render the membrane surface as hydrophilic with suitable surface modifications, which is achieved through the CNT modification process. The established data are in the good accordance with the literature [4,42,43,44]. The authors used anodic aluminium oxide membranes with nanochannels of diameters in the range of 10–120 nm and investigated the transport of water (vapor and liquid) [44] and noble gases [28] at 295 K and 1 atm. Furthermore, the impact of the modification by deposition of amorphous carbon onto Anodic Aluminum Oxide (AAO) membranes by chemical vapor deposition to reduce the hydrophobicity level was investigated. For the modified materials, a 45-fold improvement in the mass transfer of liquid water was observed in comparison to the predictions of the Hagen–Poiseuille theory of Newtonian liquids. That phenomena was attributed to the hydrodynamic slip at water–hydrophobe interfaces [45]. Moreover, the silanization reaction has been introduced to modified anodic aluminium oxide membranes [46]. An effect of surface modification on the thermal conductivity and mass transfer in membrane distillation has been studied. An important finding was the difference in thermal conductivity of membranes in the range of 1.12–1.20 Wm^−1^·K^−1^ for ceramic anodic aluminium oxide membranes, whereas standard polymeric membranes dedicated to the membrane distillation process possessed a thermal conductivity in the range of 0.04–0.08 Wm^−1^·K^−1^.

#### 3.3.2. Physicochemical Properties

**Figure 7:**
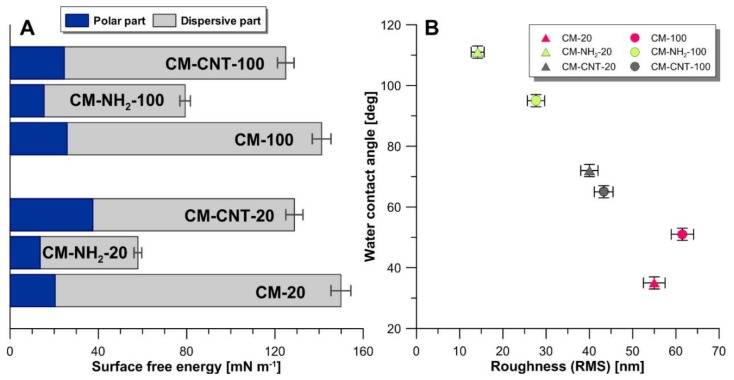
Physicochemical properties: (**A**) surface free energy with polar and dispersive components; (**B**) the relation between water contact angle (CA) and roughness parameters.

For a better understanding of the impact of functionalization on the final material properties, detailed insight into physicochemical properties was required. Therefore, the surface free energy (SFE) with its polar and dispersive components, roughness (RMS), and critical surface tension (γ_cr_) were determined. The SFE of a liquid is equal to its surface tension, and a variety of methods exist to determine it [47,48,49,50,51]. However, the surface energy of a solid cannot be measured directly and needs to be calculated from a set of solid–liquid CA tests on the solid surface. The specific surface interactions, surface reactivity, and surface solubility should also be taken into account. The applied methodologies correlate CA measurements of various liquids—with different polarities—to the SFE [48,50,52]. In the current work, the Owens−Wendt approach was implemented for the evaluation of the SFE. On the basis of this approach, the SFE is comprised of two parts: a dispersive and a polar component [47,48]. The membrane with a smaller pore size (20 nm) possessed a slightly higher SFE than those with larger pore sizes (100 nm) (Figure 7). In the silanized membranes CM-NH_2_-20 and CM-NH_2_-100, the SFEs decreased by 60% and 40%, respectively. In both of these hydrophobic membranes, the contribution by the polar component was relatively lower than the dispersive component. This observation is in agreement with the literature, wherein samples possessing hydrophobic or super-hydrophobic properties were characterized by a relatively smaller contribution of the polar component to the total value of the SFE [41,52,53,54]. The SFEs of CNT functionalized membranes, CM-CNT-20 and CM-CNT-100, were lower in comparison with the pristine membranes (Figure 7A). However, the lowest SFE values were observed in the silanized membranes (Figure 7A). The functionalization of the membranes, wherein hydrophilic CNTs were grafted onto the silanized membranes, resulted in an increase in the SFE and a significant increase in the polar component. The polar components of the SFE increased from 13.6 to 37.5 mN·m^−1^ for the 20 nm membrane and from 15.4 to 24.6 mN·m^−1^ for the 100 nm membrane. 

**Figure 8:**
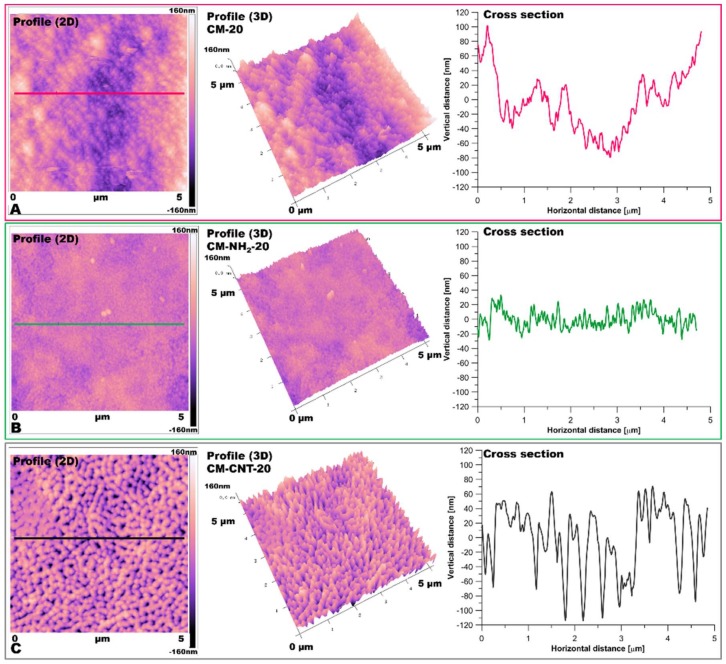
AFM imaging of membrane 2D and 3D topography and cross-section of samples: (**A**) pristine (CM-20); (**B**) after first stage of functionalization with amine groups (CM-NH_2_-20); (**C**) after second stage of functionalization with CNT (CM-CNT-20).

**Figure 9:**
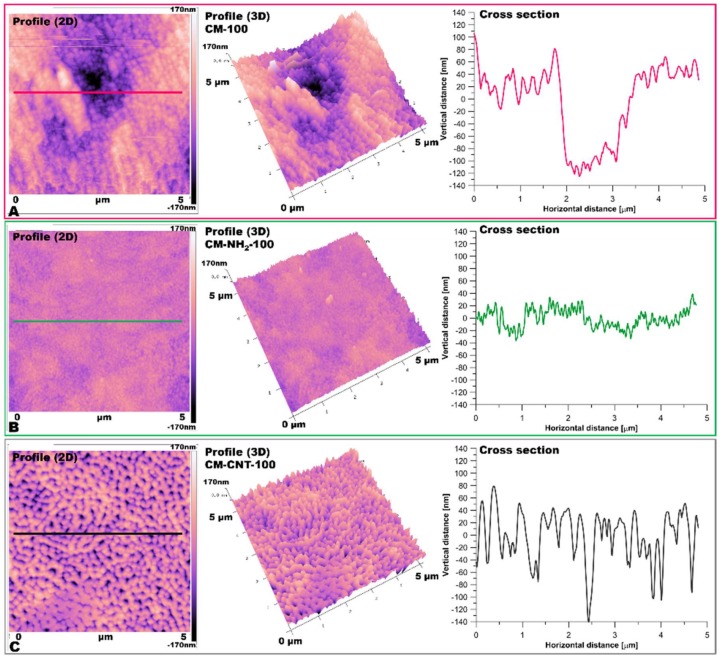
AFM imaging of membrane 2D and 3D topography and cross-section of samples: (**A**) pristine (CM-100); (**B**) after first stage of functionalization with anime groups (CM-NH_2_-100); (**C**) after second stage of functionalization with CNT (CM-CNT-100).

Utilizing AFM measurements (Figure 8 and Figure 9), roughness and surface geometry parameters were expressed as a RMS average of height deviations taken from the mean data plane. The RMS parameter was obtained on the basis of the built-in mathematical algorithm in Gwyddion 2.45 Software (Freeware version). The RMS value is a suitable parameter to examine progressive changes caused by the creation of new surfaces as well as spatial modifications as an impact of functionalization. The alteration in sample topography is presented in Figure 8 and Figure 9. The 2D and 3D profiles and cross-sections of chosen samples clearly indicate the high influence of the functionalization process on the membrane micro-architecture. The RMS is very sensitive to large deviations with respect to the mean line [41,55]. After silanization, the roughness decreased from 55 ± 2.5 to 14 ± 1.5 nm for the CM-NH_2_-20 membrane (Figure 8) and from 62 ± 2.6 to 28 ± 2.0 nm for the CM-NH_2_-100 membrane (Figure 8). However, after CNT functionalization, the roughness of the membranes increased, as is evident in the SEM images (Figure 8D). The RMS values increased to 40 ± 2.1 and 43 ± 2.2 nm for CM-CNT-20 and CM-CNT-100, respectively. The increase in the surface roughness for samples containing CNTs could be correlated to the rigid cylindrical structure of the CNTs and bundle formation. On the basis of Figure 7B, it can be deduced that there was a strong correlation between the water CA and the roughness of the membranes. As the surface roughness increased, the water CA decreased.

**Figure 10:**
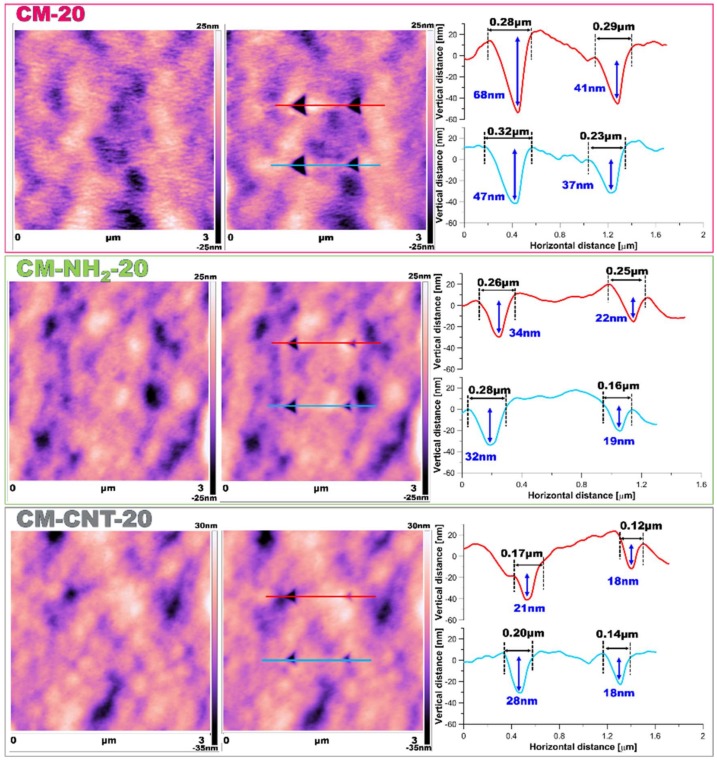
Nano-indentation test for pristine and modified samples of 20 nm membranes.

**Figure 11:**
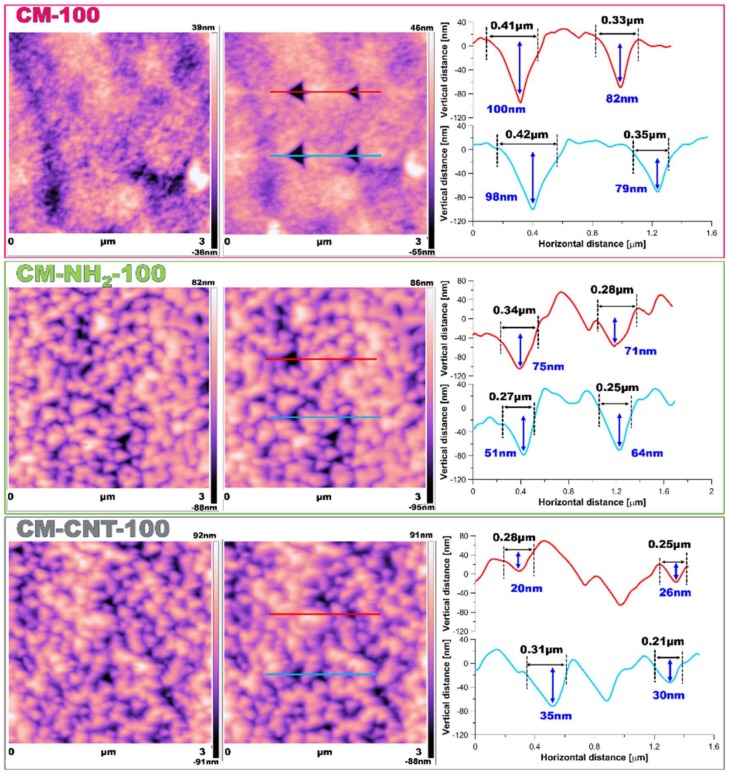
Nano-indentation test for pristine and modified samples of 100 nm membranes.

The mechanical strength of all membranes tested in this work was assessed by tribological measurements using an AFM technique utilizing the adhesion force (F_adh_), the nanohardness (H), and Young’s modulus (E). The results are shown in Table 1. AFM analysis for nano-indentation was conducted and the results are presented in Figure 10 (for 20 nm membrane) and Figure 11 (for 100 nm membrane). Generally, higher values were achieved after each modification step.

As a consequence of CNT grafting, both Young’s modulus and the nanohardness of the membranes increased, indicating better mechanical and tribological properties. The nanohardness also changed considerably (≈80%) for both the CM-CNT-20 and CM-CNT-100 membranes. However, the nanohardness values were relatively lower for the 100 nm membranes, indicating a less-dense structure of these membranes (Table 1). Nonetheless, in general, the mechanical and tribological properties were improved after grafting with CNTs.

**Figure 12:**
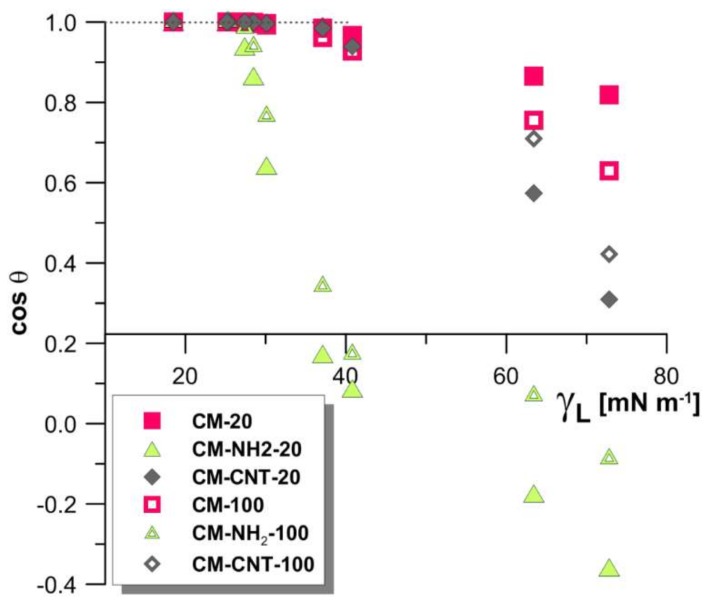
Zisman plot of pristine and modified membranes.

Critical surface tension values of the pristine and modified membranes were determined according to Zisman’s method by plotting the cosine of the CA against the surface tension of a variety of liquids (values and order mentioned in Section 2.3) [56]. The critical surface tension of the pristine membranes was higher compared to the modified membranes (Figure 12). The values of γ_cr_ were 31.8 and 30.1 mN·m^−1^ for CM-20 and CM-100, respectively. As these values are comparable, it is understood that the variation in the pore size of the membranes did not have a large impact on the critical surface tension. After silanization, the values of γ_cr_ decreased to 24.0 and 27.3 mN·m^−1^ for CM-NH_2_-20 and CM-NH_2_-100, respectively. These results explain the observed water-resistant properties of the silanized membranes. With the subsequent CNT functionalization, values of γ_cr_ increased up to 30.0 and 32.1 mN·m^−1^ for CM-CNT-20 and CM-CNT-100, respectively.

**Figure 13:**
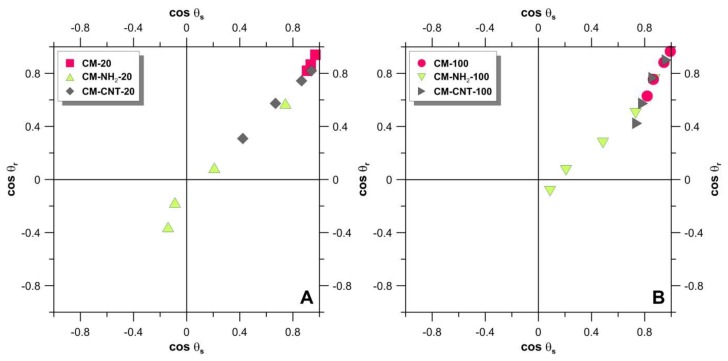
Kao diagram of 20 nm (**A**) and 100 nm (**B**) membranes as pristine, silanized, and single-wall carbon nanotube (SWCNT) functionalized material; solvents from left to right for each membrane are water, glycerol, 1-methyl-2-pyrolidone, and *N*,*N*-dimethyl-formamide.

Another powerful tool to combine the hydrophobicity/hydrophilicity of the surface with homogeneity/heterogeneity, as well as with wettability properties, is a Kao diagram [41,53,57]. Utilizing the CA measurements of a wide range of wetting liquids with varying liquid surface tension, it was possible to compare the physicochemical properties of the modified membranes in terms of wettability and surface roughness simultaneously (Figure 13).

The first quadrant of the coordinate system in Figure 13 (upper right) represents hydrophilic and super-hydrophilic surfaces with low roughness values. The third quadrant (lower left) represents rough surfaces with a hydrophobic character (Cassie–Baxter’s region), which describes wettability on heterogeneous surfaces [53,57,58,59]. Between these two areas Wenzel’s region is located, combining a hydrophobic character and wettability with homogeneous surfaces [53,57,58,59]. Figure 13 shows that in the case of liquids with a low surface tension (i.e., toluene, tetrahydrofuran, and n-dodecane), material structure wetting was observed for all membranes. On the other hand, in the case of high-surface-tension liquids (e.g., water), only pristine membranes (CM-20 and CM-100) showed wetting (Figure 13). Hydrophobic surfaces formed after salinization were found in Wenzel’s region of the Kao diagram. As a result of the silanization process, a reduction in surface heterogeneity and an increase in hydrophobicity (CA of 111° for CM-NH_2_-20 and 95° for CM-NH_2_-100) were observed. The CM-CNT-20 and CM-CNT-100 membranes were located in Wenzel’s region. This was associated with a substantial drop in the hydrophobicity level by the grafting of CNTs. Despite the reduction in the CA, a growth in heterogeneity was observed, and the samples maintained their water-resistance property because of the presence of a silane layer between the raw ceramic and SWCNTs.

It can be observed from Figure 13 that the grafting of the membranes with T-NH_2_ and subsequent chemical anchoring of CNTs clearly changed the physicochemical properties and morphology of the ceramic membranes.

## 4. Conclusions

The development of advanced functional membranes with tailored properties is key to exploiting the potentialities of membrane-based separation processes. An important approach toward this aim is the realization of composite systems in which organic and inorganic phases coexist in order to have synergic effects on membrane properties. In this study, the surfaces of nano-ordered ceramic membranes were covalently functionalized by amino silanization and were further grafted with SWCNTs on the surface. The resultant inorganic–organic hybrid membrane integrates the organic chemical functionality towards selectivity with transport efficiency through the inorganic neat hierarchy. This approach possesses many advantages compared with the methods presented in the scientific literature. Our method is characterized by simplicity and high effectiveness. The modified membranes had improved water resistance and possessed better mechanical properties. Physicochemical properties such as hydrophobicity, surface free energy, and tribological properties of the developed membranes were also significantly improved. The covalent attachment of CNTs onto the ceramic membranes was demonstrated by HR-TEM analysis and Raman spectroscopy. The grafted CNTs could be found on the surface of the membrane as well as partially inside the pores. Moreover, SAED analysis confirmed the increase in crystallinity, with a distance of around 0.30 nm between the nanotubes. Dynamic goniometric measurements were used to evaluate the hydrophobicity, surface free energy, and wetting properties. Silanization resulted in creating waterproof surfaces with CAs increasing from 38° to 110° and from 51° to 95° for the CM-NH_2_-20 and CM-NH_2_-100 membranes, respectively. SWCNT functionalization reduced the CA to 72° and 66° for CM-CNT-20 and CM-CNT-100, respectively. Modifications also influenced the mechanical properties of the formed hybrid materials. From the nanotribological study, Young’s modulus increased from 33 to 39 GPa for CM-CNT-20 and from 43 to 48 GPa for CM-CNT-100 in comparison with the pristine membranes. Furthermore, the nanohardness increased by about 80% after the attachment of CNTs for both membranes. A direct correlation of surface chemistry and surface roughness of the membranes was demonstrated using a Kao diagram. Membrane wetting resistance data showed that both amine-functionalized and CNT-bound membranes possessed a higher water resistance compared to the pristine ceramic membrane. The Zisman plot for the pristine and modified membranes also showed that both amine functionalization and grafting with CNTs caused a diminution in the critical surface tension (γ_cr_), indicating a higher wetting resistance of CM-NH_2_ and CM-CNT membranes compared to the pristine ceramic support.

## Figures and Tables

**Table 1:** Tribological characterization of pristine and functionalized ceramic membranes.

Sample	Adhesion Force (nN)	Nanohardness (GPa)	Young’s Modulus (GPa)
CM-20	12.6 ± 0.9	1.23 ± 0.09	33.08 ± 2.32
CM-NH_2_-20	14.8 ± 1.0	5.68 ± 0.40	35.09 ± 2.46
CM-CNT-20	18.5 ± 1.3	6.62 ± 0.46	39.14 ± 2.74
CM-100	8.3 ± 0.6	0.45 ± 0.03	42.86 ± 3.00
CM-NH_2_-100	10.8 ± 0.8	2.04 ± 0.13	45.38 ± 3.18
CM-CNT-100	15.7 ± 1.1	2.34 ± 0.16	48.67 ± 3.41
